# Association of lactase persistence genotype with milk consumption, obesity and blood pressure: a Mendelian randomization study in the 1982 Pelotas (Brazil) Birth Cohort, with a systematic review and meta-analysis

**DOI:** 10.1093/ije/dyw074

**Published:** 2016-05-11

**Authors:** Fernando Pires Hartwig, Bernardo Lessa Horta, George Davey Smith, Christian Loret de Mola, Cesar Gomes Victora

**Affiliations:** 1 Postgraduate Program in Epidemiology, Federal University of Pelotas, Pelotas, Brazil; 2 MRC Integrative Epidemiology Unit, University of Bristol, Bristol, UK

**Keywords:** Lactase persistence, milk, body mass index, obesity, blood pressure, Mendelian randomization

## Abstract

**Background:** Milk intake has been associated with lower blood pressure (BP) in observational studies, and randomized controlled trials suggested that milk-derived tripeptides have BP-lowering effects. Milk intake has also been associated with body mass index (BMI). Nevertheless, it is unclear whether increasing milk consumption would reduce BP in the general population.

**Methods:** We investigated the association of milk intake with obesity and BP using genetically-defined lactase persistence (LP) based on the rs4988235 polymorphism in a Mendelian randomization design in the 1982 Pelotas (Southern Brazil) Birth Cohort. These results were combined with published reports identified through a systematic review using meta-analysis.

**Results:** In the 1982 Pelotas Birth Cohort, milk intake was 42 [95% confidence interval (CI): 18; 67) ml/day higher in LP individuals. In conventional observational analysis, each 1-dl/day increase in milk intake was associated with −0.26 (95% CI: −0.33; −0.19) kg/m^2^ in BMI and −0.31 (95% CI: −0.46; −0.16) and -0.35 (95% CI: −0.46; −0.23) mmHg in systolic and diastolic BP, respectively. These results were not corroborated when analysing LP status, but confidence intervals were large. In random effects meta-analysis, LP individuals presented higher BMI [0.17 (95% CI: 0.07; 0.27) kg/m^2^] and higher odds of overweight-obesity [1.09 (95% CI: 1.02; 1.17)]. There were no reliable associations for BP.

**Conclusions:** Our study supports that LP is positively associated with obesity, suggesting that the negative association of milk intake with obesity is likely due to limitations of conventional observational studies. Our findings also do not support that increased milk intake leads to lower BP.

Key Messages

- Genetically defined lactase persistence (LP) was associated with more milk intake in the 1982 Pelotas Birth Cohort, and this association was more pronounced among individuals of more European ancestry.
- Milk intake was negatively associated with obesity and blood pressure in conventional observational analysis in the 1982 Pelotas Birth Cohort.
- LP was positively associated with BMI and overweight-obesity in our meta-analysis which was based on a systematic literature review.
- There were no reliable meta-analytical associations between LP and blood pressure, thus not supporting the notion that milk intake is causally associated with lower blood pressure.

## Introduction

Single nucleotide polymorphisms (SNPs) in the enhancer region of the *LCT* gene [OMIM: 603202] are functionally associated with adult-type hypolactasia;^1^ rs4988235 (a C to T SNP located 13910 base pairs downstream of the *LCT* gene) is one such SNP, whose association with lactase persistence (LP)—maintenance of lactase expression after weaning [MIM: 223100]—was first identified in Finland.^2^ The presence of the T allele (i.e. CT and TT genotypes) correlated perfectly with LP, which is considered an autosomal dominant trait^3^ even though the effects at the gene expression level are additive.^4^^,^^5^ This association was corroborated by several functional studies^4^^,^^6–9^ which revealed the causal effect of rs4988235 on LP. Further genetic variability in this region has been described in non-Europeans, including admixed populations such as Brazil.^10^ The gastrointestinal symptoms in response to lactose that occur (at varying levels) in individuals with adult-type hypolactasia motivated investigation of the positive association between LP and intake of milk and other dairy products.^2^^,^^11^^,^^12^ This association was observed in several populations^13–18^ but not universally, possibly for cultural reasons involved with acceptance and generality of milk drinking.^19^^,^^20^

Cardiovascular diseases are the leading cause of death worldwide. Ischaemic heart disease and stroke were estimated to have caused around 25% of deaths in the world in 2010.^21^ Nevertheless, much of this burden is considered preventable. For example, the risk of developing coronary heart disease was 83% lower in women presenting a healthy lifestyle in a prospective study (14 years of follow-up) of approximately 80 000 healthy (at baseline) participants in the Nurses’ Health Study.^22^ Among many aspects of diet, intake of milk and other dairy products has been suggested to protect against cardiovascular diseases through a blood pressure-lowering effect. Associations of milk with lower risk of hypertension have been reported in several observational studies.^23^^,^^24^ In addition, randomized controlled trials (RCTs) indicate that specific milk components may lower blood pressure.^25^^,^^26^

Some studies also detected associations between dairy consumption and body mass index (BMI), but the literature is controversial. Observational studies in children reported positive associations between milk consumption and BMI, especially in children of 4 years of age or younger.^27^^,^^28^ However, a meta-analysis of 22 studies failed to detect such association, although a negative association between dairy intake and adiposity in adolescents was detected.^29^ In adults, a prospective study in almost 100 000 Danish individuals identified a positive association between intake of any type of milk and risk of overweight-obesity; however, there was no dose-response relationship, and the association was observed for fat-free, but not for high-fat, milk.^30^ Moreover, replacing water with milk was associated with increased total energy intake in a systematic review of intervention studies, although these findings were not considered conclusive.^31^ Findings from three cohort studies with a total sample size of more than 120 000 adults failed to detect an association between whole- and low-fat milk intake and prospective weight gain, in multivariable analysis. However, individuals who replaced sugar-sweetened beverages and fruit juices with milk presented lower prospective weight gain than their counterparts,^32^ suggesting the association between milk consumption and BMI might be influenced by overall dietary patterns. Therefore, the direction of the association (if any) of milk intake with obesity is unclear.

The use of genetic variants as proxies of modifiable exposures can improve causal inference in observational studies through applying the principle of Mendelian randomization.^33^ Appropriately performed, Mendelian randomization studies are not prone to confounding or reverse causation and, given that its assumptions (i.e. no confounding by population stratification or by horizontal pleiotropy) are not violated, provide a strategy to obtain causal effect estimates in observational studies.^34,35^

Associations involving LP status and cardiovascular risk factors have already been reported in the literature. A meta-analysis involving 31 720 Europeans reported that T-allele carriers (i.e. LP individuals) had higher BMI than CC individuals (i.e. non-persistents).^36^ Given that genetically-defined LP has been associated with higher milk intake in these populations,^13–18^ the association between LP and BMI may be due to differences in milk consumption. However, the aforementioned large Danish study did not detect strong associations with obesity and blood pressure.^30^^,^^37^

In the present study, we evaluated whether genetically-defined LP is associated with milk intake, obesity and blood pressure among subjects who have been followed up since birth in a southern Brazilian city. We also combined these results with published data identified through a systematic review of the literature.

## Methods

### 1982 Pelotas Birth Cohort

#### Study participants and data collection

In 1982, the maternity hospitals in Pelotas, a southern Brazilian city (current population 330 000), were visited daily by trained interviewers, who were health professionals with an academic degree. The 5914 liveborns whose families lived in the urban area were examined and their mothers interviewed. We were able to get information of more than 99% of the live births. These subjects have been followed-up nine times (five follow-ups targeting subsamples and four targeting the whole cohort). From October 2004 to August 2005, we visited all households located in the urban area of the city. For those not located in this way we used the last known address. Subjects (aged 22–23 years) were interviewed and examined at home and invited to visit the research laboratory to donate a blood sample, collected by venous puncture. DNA and sera were extracted and frozen at −70°C. DNA samples were genotyped using the Illumina HumanOmni2.5-8v1 array.^38^^,^^39^ From June 2012 to February 2013, all cohort participants (aged 30–31 years) were invited to visit the research clinic to be interviewed and examined.^39^ A total of 3701 subjects were interviewed in the 2012–13 follow-up visit. Taking into account the 325 individuals who are known to have died, this represents a follow-up rate of 68.1%. Among these, 2843 (comprising the group included in the present study) had data for the rs4988235 SNP and at least one studied outcome (i.e. BMI, systolic or diastolic blood pressure) (Supplementary Figure 1, available as Supplementary data at *IJE* online).

Exposure variables were rs4988235 SNP and dairy intake. We selected the rs4988235 SNP as a potential genetic proxy of milk consumption due to its role in LP.^2^^,^^7–9^ Daily dairy consumption (milk and yogurt in ml/day, and cheese and cottage cheese in g/day) was measured using a food frequency questionnaire with a 1-year recall in the 2012–13 follow-up (when individuals were 30–31 years of age).

Outcome variables were: BMI, overweight-obesity, systolic blood pressure, diastolic blood pressure and raised blood pressure. For BMI (kg/m^2^), weight and height were measured using portable weighing devices and stadiometers of 100 g and 1 cm of precision, respectively. Overweight-obesity was defined as BMI ≥ 25 kg/m^2^. Systolic and diastolic blood pressures were measured in mmHg using an Omron HEM-705CPINT digital sphygmomanometer; two measurements were taken and their mean was used for the analyses. Raised blood pressure was defined as systolic BP ≥ 130 mmHg or diastolic BP ≥ 85 mmHg. These variables were measured in the 2012–13 follow-up.

Covariate variables were: sex, maternal schooling at birth (in complete years of education of the mothers), family income at birth, birthweight and gestational age based on the maternal recall of the date of the last menstrual period (measured at birth); self-reported skin colour, genomic ancestry (European, African and Native American) and leisure-time physical activity (using the International Physical Activity Questionnaire) (measured at the 2004–04 visit, when individuals were 22–23 years of age); and household asset index, achieved schooling in complete years, smoking, alcohol intake, low-density lipoprotein (LDL) and height (measured in the 2012–13 visit, when individuals were 30–31 years of age). Household asset index was obtained by applying principal component analysis to a list of 12 assets and the schooling of the household head.^40^ Genomic ancestry was estimated using ADMIXTURE^41^ based on ∼370 000 SNPs mutually available for the 1982 Pelotas Birth Cohort and reference panels from the HapMap and Human Genome Diversity projects, as described elsewhere.^42^

#### Statistical analysis

Hardy-Weinberg equilibrium (HWE) and distribution of rs4988235 genotypes according to observed skin colour were evaluated using Fisher’s exact and χ^2^ tests, respectively. Unadjusted associations of milk intake status (none vs any) with sociodemographic, perinatal, lifestyle and biological variables were evaluated using the χ^2^ test (for categorical variables) or the t test (for continuous variables). The remaining analyses were performed by linear or logistic regression. To account for population stratification, estimates were adjusted for quantitative indicators of genomic ancestry (European, African and Native American) when indicated. Two genetic effect models were used: codominant (i.e. each genotype is coded as a distinct category) and dominant (i.e. CC = 0; CT or TT = 1). The codominant effect is the generic model, whereas the dominant effect is the most strongly supported by the literature regarding the consequences of lactase non-persistence at the distal phenotypic level (e.g. on milk intake).^3^

### Systematic review and meta-analysis

A systematic review of the literature regarding the association of LP with obesity and blood pressure was performed through Ovid [https://ovidsp.tx.ovid.com/] which allows simultaneously searching the following databases: MEDLINE, Embase, Allied and Complementary Medicine Database, CAB ABSTRACTS, PsycINFO^®^ and The Philosopher’s Index. The following combination of search terms was used: ‘LP’ AND (‘BMI’ OR ‘Blood pressure’ OR ‘Other’). A detailed description of each search term, as well as the number of records retrieved by each in isolation, is provided in Supplementary Tables 1 and 2, respectively (available as Supplementary data at *IJE* online). The resulting records were independently evaluated by two reviewers, and disagreements were resolved by consensus.

Studies that analysed the association of the rs4988235 variant (exposure) with the following outcomes: BMI (continuous), overweight-obesity (binary), systolic and diastolic blood pressure (continuous) and raised blood pressure/hypertension (binary) were included. Exclusion criteria were: (i) unavailability of results for the dominant model (i.e. comparing CC individuals with T-allele carriers) or sufficient data for its calculation; and (ii) articles not reporting original data.

The following characteristics of the included studies were extracted:

- first author’s name;
- year of publication;
- country and continent where the sample was taken;
- ancestry (European, African, Hispanic, Asian, Other [e.g. admixed samples]);
- sample size;
- prevalence of genetically defined LP;
- mean age;
- study design (unrelated individuals or family-based);
- ascertainment for a given phenotype [0 = no; 1 = yes (e.g. controls and cases, respectively, in a case-control study)];
- cut-off used to determine overweight-obesity or raised blood pressure/hypertension;
- covariates adjusted for in multivariable models. After data extraction, the following variables were generated to indicate covariates adjusted for in individual studies: sex (either sex-adjusted or sex-specific), age (either age-adjusted or age-specific) and population substructure (birthplace coordinates, region of residence, ethnic group, genetic admixture, etc).
- mean differences for continuous outcomes, and logistic regression coefficients (and associated standard errors) for binary outcomes (either directly from the publications or calculated based on data available) referring to a comparison between CC individuals (reference) and T-allele carriers (i.e. CT or TT individuals).

For some studies^13^^,^^16^^,^^19^^,^^30^^,^^43–52^ it was necessary to calculate the standard error of the mean difference (SE). This was performed as follows:

$$\text{SE = }\sqrt{{\text{s}}_{\text{CC}}^{\text{2}}{\text{/n}}_{\text{CC}}{\text{+s}}_{\text{LP}}^{\text{2}}{\text{/n}}_{\text{LP}}}\text{,}$$

where and s_i_ and n_i_ are the standard deviation and sample size, respectively, for non-LP (i = CC) and LP (i = LP) individuals.

For some studies,^16^^,^^19^^,^^30^^,^^47^^,^^50^ it was necessary to combine the data for CT and TT individuals to obtain mean and standard deviations for T-carriers (or LP individuals). The mean values were pooled as follows:

$$\mu_{\text{LP}}\text{ = }\sqrt{\left({\text{n}}_{\text{CT}}{\times \mu }_{\text{CT}}^{\text{2}}{\text{+n}}_{\text{TT}}{\times \mu }_{\text{TT}}^{\text{2}}\right)\text{/}\left({\text{n}}_{\text{CT}}{\text{+n}}_{\text{TT}}\right)},$$

where *μ*_LP_ is the combined mean, and n_i_ and *μ*_i_ are the sample size and mean, respectively, for CT (i = CT) and TT (i = TT) individuals.

Standard deviations were pooled as follows:

$${\text{ s}}_{\text{LP}}\text{ = }\sqrt{\left[\left({\text{n}}_{\text{CT}}-\text{1}\right){\text{s}}_{\text{CT}}^{\text{2}}\text{+}\left({\text{n}}_{\text{TT}}-\text{1}\right){\text{s}}_{\text{TT}}^{\text{2}}\right]\text{/}\left({\text{n}}_{\text{CT}}{\text{+n}}_{\text{TT}}\right)}$$

where s_LP_ is the combined standard deviation, and n_i_ and s_i_ are the sample size and standard deviation, respectively, for CT (i = CT) and TT (i = TT) individuals.

When only the median and interquartile range were available,^30^^,^^49^ the outcome was assumed to be approximately normally distributed, so the median was used as an estimate of the mean. Assuming a normal distribution also allows estimating the standard deviation, because the interval from -0.674 to + 0.674 standard deviations would be expected to contain 50% of the observations around the mean. Therefore, the standard deviation was estimated by dividing the interquartile range by 2 × 0.674. Given the need to use the median as a proxy of the mean and that skewness of BMI and systolic and diastolic blood pressures were, respectively, 1.10, 0.61 and 0.55 (*P* < 0.001 in all cases) in the 1982 Pelotas Birth Cohort, the resulting standard deviation estimate was multiplied by 1.1 (i.e. a 10% increment) to avoid its underestimation due to deviations from the normal distribution.

For binary outcomes, combining CT and TT into a single group and/or estimating the necessary statistics was performed when necessary,^30^^,^^44^^,^^48^^,^^50^^,^^53^ based on number of individuals belonging to each outcome-LP status combination, as presented in the publications.

For meta-analysis, pooled effect estimates were generated using random effects models. We opted to use random effects only due to differences in characteristics of the included studies (displayed in Supplementary Data File, available as Supplementary data at *IJE* online) and to substantial between-study heterogeneity observed in three studied outcomes. The following variables were explored as heterogeneity sources in stratified analyses and random effects meta-regression models: adjustment for at least sex, age or population substructure, as well as adjustment for each of these covariates individually. The following study characteristics were also evaluated: sample size, mean age, ancestry, continent and prevalence of LP. Publication bias was evaluated through funnel plots and Egger’s regression. Analyses of influence were performed as additional sensitivity analyses by obtaining estimates excluding all studies, one at a time.

### Power calculations

*Post hoc* power calculations through simulations were performed to evaluate our meta-analysis power to detect associations of LP with systolic and diastolic blood pressure assuming that they are entirely mediated by BMI. Estimates were obtained from the aforementioned large Danish study^30^—the largest study included in our meta-analysis—and two Mendelian randomization studies.^54^^,^^55^ A detailed description of the simulations is provided in Supplementary Methods (available as Supplementary data at *IJE* online).

All analyses and simulations were performed using R version 3.2.0 [http://www.r-project.org/].

## Results

### 1982 Pelotas Birth Cohort

Supplementary Table 3 (available as Supplementary data at *IJE* online) describes the studied individuals according to sociodemographic, perinatal, lifestyle and biological variables. Compared with non-drinkers, milk drinkers presented a higher proportion of males, maternal schooling, family income at birth, birthweight, gestational age, proportion of self-reported White skin colour, European genomic ancestry, achieved schooling, household asset index, height and yogurt and cottage cheese intake. Inverse associations were observed for African and Native American genomic ancestry, BMI and diastolic blood pressure.

A comparison of baseline characteristics of the whole cohort and of the entire 2012–13 years-of-age follow-up visit, with those included in the present study, is available in Supplementary Table 4 (available as Supplementary data at *IJE* online); 54% of the whole cohort and 77% of the 2012–13 follow-up were included. Compared with the whole cohort, studied individuals presented lower proportions of males and of wealthier socioeconomic positions, although there were no differences regarding birthweight. Compared with the 2012–13 follow-up, studied subjects presented higher prevalence of obesity (BMI ≥ 30 kg/m^2^).

In general, dairy consumption was positively associated with socioeconomic status (Supplementary Table 5, available as Supplementary data at *IJE* online). The prevalence of the lactase non-persistence genotype (CC) was higher among self-reported Black (70.2%) compared with White individuals (37.3%). Such differences did not remain after controlling for genomic ancestry (Supplementary Table 6, available as Supplementary data at *IJE* online). There was no strong indication that the genotypic frequencies deviated from Hardy-Weinberg Equilibrium when evaluating all individuals [expected/observed frequencies: 43.1%/43.7% (CC genotype), 45.1%/44.1% (CT genotype) and 11.8/12.2% (TT genotype); *P* = 0.244] and stratifying by self-reported skin colour (*P* ≥ 0.700). These results corroborate the need to take ethnicity/ancestry into account in associations between rs4988235 and dairy consumption to minimize confounding due to population stratification.

Table 1 shows that milk intake was higher among T allele-carriers, whose consumption was (in adjusted models) 42 (95% CI: 18; 67) ml/day higher than CC individuals. The similarity of mean consumption values between CT and TT genotypes corroborates that the T-allele of the rs4988235 SNP has a dominant effect on this outcome. These associations differed between strata of European genomic ancestry, with estimates of 73 (95%: 38; 108) and 12 (95% CI: -21; 46) in individuals with European genomic ancestry≥85% and < 85%, respectively (interaction test *P*-value = 0.021). Reliable associations were not observed for any other dairy product.

**Table 1.** Dairy intake in the 1982 Pelotas (Brazil) Birth Cohort according to rs4988235. Values are number of individuals (*N*), means of daily intake (95% confidence intervals), R^2^-values, F-statistics (F) and *P*-values (*P*)

Dairy product	Genetic model	Unadjusted	Adjusted for sex and genomic ancestry		
			All	European^a^	Other^b^
		(*N* = 2808)	(*N* = 2806)	(*N* = 1436)	(*N* = 1370)
Milk (ml)	**Codominant**			P_interaction_ = 0.063	
		*P* = 1.5 × 10^−4^	*P* = 0.003	*P* = 2.6 × 10^−4^	*P* = 0.740
	C/C	0 (Ref.)	0 (Ref.)	0 (Ref.)	0 (Ref.)
	C/T	50 (26; 75)	43 (18; 69)	75 (38; 113)	14 (−21; 48)
	T/T	49 (12; 86)	39 (0; 77)	67 (17; 116)	3 (−64; 70)
	**Dominant**			P_interaction_ = 0.021	
		R^2 ^= 0.63%	R^2 ^= 0.42%	R^2 ^= 1.14%	R^2 ^= 0.04%
		F = 17.71	F = 11.77	F = 16.46	F = 0.51
		*P* = 2.7 × 10^−5^	*P* = 0.001	*P* = 5.2 × 10^−5^	*P* = 0.475
	C/T or T/T	50 (27; 73)	42 (18; 67)	73 (38; 108)	12 (−21; 46)
Yogurt (ml)	Codominant			*P*_interaction_ = 0.644	
		*P* = 0.162	*P* = 0.193	*P* = 0.096	*P* = 0.868
	C/C	0 (Ref.)	0 (Ref.)	0 (Ref.)	0 (Ref.)
	C/T	13 (0; 25)	12 (−1; 26)	19 (2; 36)	6 (−15; 26)
	T/T	5 (−15; 24)	4 (−16; 25)	7 (−15; 30)	4 (−36; 44)
	Dominant			*P*_interaction_ = 0.454	
		R^2 ^= 0.11%	R^2 ^= 0.09%	R^2 ^= 0.25%	R^2 ^= 0.02%
		F = 3.00	F = 2.65	F = 3.62	F = 0.28
		*P* = 0.083	*P* = 0.104	*P* = 0.057	*P* = 0.599
	C/T or T/T	11 (−1; 23)	11 (−2; 23)	16 (0; 32)	5 (−15; 25)
Cheese (g)	Codominant			P_interaction_ = 0.658	
		*P* = 0.059	*P* = 0.035	*P* = 0.105	*P* = 0.223
	C/C	0 (Ref.)	0 (Ref.)	0 (Ref.)	0 (Ref.)
	C/T	0.6 (−0.8; 2.0)	0.2 (−1.2; 1.7)	0.7 (−1.2; 2.6)	−0.1 (−2.2; 2.0)
	T/T	−1.9 (−4.0; 0.2)	−2.5 (−4.7; −0.3)	−1.9 (−4.4; 0.6)	−3.6 (−7.7; 0.6)
	Dominant			P_interaction_ = 0.578	
		R^2 ^= 0.00%	R^2 ^= 0.01%	R^2 ^= 0.00%	R^2 ^= 0.02%
		F = 0.02	F = 0.20	F = 0.00	F = 0.30
		*P* = 0.902	*P* = 0.656	*P* = 0.988	*P* = 0.585
	C/T or T/T	0.1 (−1.2; 1.4)	−0.3 (−1.7; 1.1)	0.0 (−1.8; 1.8)	−0.6 (−2.6; 1.5)
Cottage	Codominant			*P*_interaction_ = 0.859	
cheese (g)		*P* = 0.971	*P* = 0.587	*P* = 0.689	*P* = 0.928
	C/C	0 (Ref.)	0 (Ref.)	0 (Ref.)	0 (Ref.)
	C/T	0.1 (−0.5; 0.6)	−0.2 (−0.8; 0.3)	−0.3 (−1.2; 0.5)	−0.1 (−0.8; 0.6)
	T/T	0.1 (−0.7; 0.9)	−0.4 (−1.2; 0.5)	−0.4 (−1.6; 0.8)	−0.2 (−1.6; 1.1)
	Dominant			P_interaction_ = 0.558	
		R^2 ^= 0.00%	R^2 ^= 0.03%	R^2 ^= 0.05%	R^2 ^= 0.01%
		F = 0.05	F = 0.96	F = 0.73	F = 0.12
		*P* = 0.820	*P* = 0.326	*P* = 0.392	*P* = 0.727
	C/T or T/T	0.1 (−0.5; 0.6)	−0.3 (−0.8; 0.3)	−0.4 (−1.2; 0.5)	−0.1 (−0.8; 0.5)

^a^Individuals with European genomic ancestry ≥ 85%.

^b^Individuals with European genomic ancestry < 85%.

Milk intake was inversely associated with obesity and blood pressure (Table 2). After controlling for sociodemographic variables (which did not substantially influence the results), each increment of 1 dl/day was associated with 0.26 (0.19; 0.33) kg/m^2^ lower BMI, 0.31 (95% CI: 0.16; 0.46) and 0.35 (95% CI: 0.23; 0.46) mmHg lower systolic and diastolic blood pressure, respectively, as well as with odds ratio of 0.93 (95% CI: 0.91; 0.96) and 0.93 (95% CI: 0.90; 0.96) for overweight-obesity and raised blood pressure, respectively. There were no reliable associations supporting effect modification by sex (*P* > 0.20 for all outcomes) and European genomic ancestry (P = 0.12 for all outcomes).

**Table 2.** Association of milk intake (in decilitres per day) with studied outcomes in the 1982 Pelotas (Brazil) Birth Cohort. Values are number of individuals (*N*), linear regression coefficients (β) or odds ratio (OR), 95% confidence intervals (95% CI) and *P*-values (*P*)

Outcome	Unadjusted	Adjusted^a^
BMI (kg/m^2^)		
*N*	2761	2595
*P*	1.5 × 10^−13^	3.2 × 10^−13^
β (95% CI)	−0.25 (−0.32; −0.18)	−0.26 (−0.33; −0.19)
Overweight-obesity^b^		
*N*	2761	2595
*P*	1.8 × 10^−7^	7.2 × 10^−8^
OR (95% CI)	0.94 (0.91; 0.96)	0.93 (0.91; 0.96)
Systolic BP (mmHg)		
*N*	2806	2638
*P*	0.001	3.7 × 10^−5^
β (95% CI)	−0.27 (−0.44; −0.11)	−0.31 (−0.46; −0.16)
Diastolic BP (mmHg)		
*N*	2806	2638
*P*	2.0 × 10^−8^	2.9 × 10^−9^
β (95% CI)	−0.32 (−0.43; −0.21)	−0.35 (−0.46; −0.23)
Raised BP^c^		
*N*	2806	2638
*P*	2.9 × 10^−4^	1.9 × 10^−5^
OR (95% CI)	0.95 (0.92; 0.98)	0.93 (0.90; 0.96)

^a^Adjusted for sex, maternal schooling at birth (0–4, 5-8, 9–11 or ≥ 12 complete years), family income at birth (≤ 1, 1.1–3, 3.1–6, 6.1–10 or > 10 minimum wages), African and Native American genomic ancestry (continuous, as % values), household asset index at 30–31 years of age in quintiles and achieved schooling at 30–31 years of age (0-4, 5–8, 9–11 or ≥ 12 complete years).

^b^BMI ≥ 25 kg/m^2^.

^c^Systolic BP ≥ 130 mmHg or diastolic BP ≥ 85 mmHg.

Supplementary Table 7 (available as Supplementary data at *IJE* online) describes the association of genetically-defined LP with potential confounders of the association of milk intake with obesity and/or blood pressure. In unadjusted analyses, LP individuals had 0.4 (95% CI: 0.1; 0.7) more complete years of education than non-LP individuals, as well as household asset index higher in 0.1 (95% CI: 0.1; 0.2) standard deviation. However, after controlling for genomic ancestry, the respective coefficients were -0.1 (95% CI: -0.5; 0.2) and 0.0 (95% CI: −0.1; 0.1). The odds of LP among current smokers was 1.4 (95% CI: 1.1; 1.6) times higher in never smokers in adjusted models. There was no strong indication that any of the reported associations differ according to milk intake status, except for alcohol intake: compared with non-LP, LP status was associated with 2.9 (95% CI: 5.5; 0.3) g/day less alcohol intake among non-milk drinkers, and 0.5 (−0.7; 1.6) g/day more among milk drinkers.

There were no strong associations of genetically defined LP with obesity or blood pressure (Table 3), although there was some indication for BMI. In unadjusted models, BMI was 0.27 (95% CI: −0.15; 0.69) kg/m^2^ higher in LP individuals. Adjusting for genomic ancestry increased the magnitude of the estimate to 0.44 (95% CI: 0.00; 0.88). These results were directionally consistent with the point odds ratio estimate for overweight-obesity [1.09 (95% CI: 0.93; 1.28)]. Moreover, point estimates of both outcomes were directionally consistent comparing unadjusted and adjusted models, as well as strata of European genomic ancestry and of milk intake status (Supplementary Table 8, available as Supplementary data at *IJE* online). For systolic, diastolic and raised blood pressure, point estimates were inconsistent comparing unadjusted with adjusted models, but all unadjusted 95% CIs included the respective adjusted point estimate and vice versa. Moreover, point estimates for systolic blood pressure and raised blood pressure were directionally inconsistent between strata of European genomic ancestry, with some indication of interaction (although 95% CIs were wide) for raised blood pressure: odds ratio of 1.20 (95% CI: 0.93; 1.55) and 0.87 (95% CI: 0.67; 1.13) in individuals with European genomic ancestry ≥ 85% and < 85%, respectively.

**Table 3.** Association of genetically defined LP (reference group: non-LP individuals) with studied outcomes in the 1982 Pelotas (Brazil) Birth Cohort. Values are number of individuals (*N*), linear regression coefficients (β) or odds ratio (OR), 95% confidence intervals (95% CI) and *P*-values (*P*)

Outcome	Unadjusted	Adjusted for sex and genomic ancestry		
		All	European^a^	Other^b^
BMI (kg/m^2^)			*P*_interaction_ = 0.467	
*N*	2782	2780	1427	1353
*P*	0.207	0.052	0.384	0.074
β (95% CI)	0.27 (−0.15; 0.69)	0.44 (0.00; 0.88)	0.26 (−0.33; 0.86)	0.60 (−0.06; 1.25)
Overweight-obesity^c^			*P*_interaction_ = 0.612	
*N*	2782	2780	1427	1353
*P*	0.565	0.294	0.799	0.259
OR (95% CI)	1.05 (0.90; 1.22)	1.09 (0.93; 1.28)	1.03 (0.82; 1.29)	1.14 (0.91; 1.43)
Systolic BP (mmHg)			*P*_interaction_ = 0.520	
*N*	2840	2838	1453	1385
*P*	0.503	0.362	0.963	0.258
β (95% CI)	−0.35 (−1.38; 0.67)	0.43 (−0.50; 1.37)	−0.03 (−1.30; 1.24)	0.79 (−0.58; 2.16)
Diastolic BP (mmHg)			*P*_interaction_ = 0.665	
*N*	2840	2838	1453	1385
*P*	0.773	0.467	0.957	0.390
β (95% CI)	−0.10 (−0.80; 0.60)	0.27 (−0.45; 0.99)	0.03 (−0.97; 1.02)	0.46 (−0.59; 1.50)
Raised BP^d^			*P*_interaction_ = 0.044	
*N*	2840	2838	1453	1385
P	0.496	0.684	0.172	0.304
OR (95% CI)	0.94 (0.80; 1.11)	1.04 (0.87; 1.25)	1.20 (0.93; 1.55)	0.87 (0.67; 1.13)

^a^Individuals with European genomic ancestry < 85%.

^b^Individuals with European genomic ancestry < 85%.

^c^BMI ≥ 25 kg/m^2^.

^d^Systolic BP ≥ 130 mmHg or diastolic BP ≥ 85 mmHg.

**Table 4.** Random effects meta-analytical linear regression coefficient (β) or odds ratio (OR) estimates of BMI, overweight-obesity, systolic, diastolic and raised blood pressure, according to genetically defined LP (reference group: non-LP individuals)

Outcome	BMI (kg/m^2^)^a^	Overweight-obesity^b^	Systolic BP (mmHg)^a^	Diastolic BP(mmHg)^a^	Raised BP^b^
Number of					
Estimates	14	5	7	7	4
Individuals	142864	102640	112440	112442	101507
Heterogeneity					
*I*^2^ (%)	83.6	8.0	55.2	0.0	52.1
τ^2^	0.035	0.001	0.445	0.0	0.016
*P*-value	1.1 × 10^-21^	0.365	0.022	0.748	0.079
Meta-analysis					
β or OR (95% CI)	0.17 (0.07; 0.27)	1.09 (1.02; 1.17)	−0.04 (−0.73; 0.64)	0.02 (0.00; 0.04)	0.98 (0.84; 1.16)
*P*-value	0.001	0.015	0.902	0.098	0.846
Egger’s regression					
Bias (95% CI)^c^	1.11 (−0.22; 2.44)	0.83 (−0.80; 2.47)	−0.16 (−1.50; 1.18)	0.25 (−0.44; 0.94)	−0.37 (−3.75; 3.01)
*P*-value	0.097	0.230	0.786	0.415	0.750
					

^a^Linear regression coefficients (β) were reported for these outcomes.

^b^Odds ratio (OR) were reported for these outcomes.

^c^Corresponds to Egger’s regression intercept, which was performed using either β or log(OR) estimates.

### Systematic review and meta-analysis

After duplicate removal, 452 records were screened based on their titles and abstracts, which yielded 30 records (shown in Sheet 1 of Supplementary Data File, available as Supplementary data at *IJE* online). After accessing the full texts, 16 records were included.^13^^,^^19^^,^^30^^,^^36^^,^^37^^,^^45–53^ One additional record^16^ was identified in the reference lists of the included records, totalling 17 records (Supplementary Figure 2, available as Supplementary data at *IJE* online). Study-level characteristics and results are displayed in Sheets 2–6 of Supplementary Data File.

Table 4 displays meta-analysis results (Supplementary Figures 3–8 present forest and funnel plots for all outcomes). BMI was 0.17 (95% CI: 0.07; 0.27) kg/m^2^ higher in LP individuals, and the pooled odds ratio for overweight-obesity was 1.09 (95% CI: 1.02; 1.17). Results for diastolic blood pressure were also suggestive of a positive association with LP, with a mean difference coefficient of 0.02 (95% CI: 0.00; 0.04) mmHg comparing LP with non-LP individuals, although the confidence intervals poorly included 0. However, this was inconsistent with the pooled point odds ratio estimate for raised blood pressure (0.98) and the pooled point mean difference for systolic blood pressure (−0.04 mmHg), with both confidence intervals largely including 1 and 0, respectively.

In influence analysis, estimates for BMI ranged from 0.08 to 0.20 and none of the 95% confidence intervals included zero. Regarding overweight-obesity, the odds ratio estimates ranged from 0.07 to 1.15 and only in one case the 95% confidence intervals included 1; in this situation, the estimate was 1.10 (95% CI: 1.00; 1.20).

Although forest plots and the *I*^2^ statistic suggested considerable heterogeneity for BMI and systolic and raised blood pressure, the number of estimates only allowed exploring potential heterogeneity sources for BMI. Excluding a single small outlying study (which reported a mean BMI difference of 5.3 kg/m^2^ in a sample size of 64 individuals) reduced the *I*^2^ statistic to 30.2% and yielded an estimate of 0.08 (95% CI: 0.04; 0.12). Such exclusion also reduced Egger’s regression intercept from 1.11 (95% CI: -0.22; 2.44)—which was suggestive of publication bias, although confidence intervals were wide—to 0.41 (95% CI: -0.27; 1.09).

Meta-analytical estimates for BMI within subgroups of study characteristics are shown in Table 5. Regarding heterogeneity, the most important characteristics were ancestry and LP prevalence, which explained about 25% and 40%, respectively, of the heterogeneity in the estimates. When comparing unadjusted with adjusted estimates, the pooled effect estimates were considerably larger in unadjusted studies regardless of the covariate. After excluding the aforementioned outlying study, adjusting for any covariate and adjusting for population substructure were the factors that explained most of the heterogeneity (about 65% and 60%, respectively). However, all pooled unadjusted estimates were larger than the corresponding adjusted estimates, regardless of the covariate (Table 6).

**Table 5.** Random effects meta-analysis and meta-regression results for the mean difference (β) in BMI comparing LP with non-LP (reference group) individuals, stratifying according to study characteristics

Characteristic	Number of		Meta-analysis		Meta-regression	
	Estimates	Individuals	β (95% CI)	*P*-value	Adjusted-R^2^	*P*-value
Sample size						
64-529	10	3518	0.50 (−0.75; 1.76)	0.431	3.81	0.300
580–2104	9	11788	0.13 (−0.02; 0.28)	0.081		
2126–97811	9	127558	0.08 (0.05; 0.11)	7.1 × 10^-7^		
Age						
9–13	3	1470	0.14 (−0.29; 0.56)	0.529	0.00	0.970
26–55	16	28611	0.24 (0.08; 0.40)	0.004		
56–69	9	112783	0.10 (0.03; 0.18)	0.006		
Ancestry						
European	25	141153	0.08 (0.04; 0.12)	3.7 × 10^-4^	24.62	0.010
Other	3	1711	1.92 (−1.02; 4.86)	0.201		
Continent						
European	23	139392	0.08 (0.03; 0.12)	4.8 × 10^-4^	14.68	0.058
Other	5	3472	1.12 (−0.61; 2.86)	0.204		
LP prevalence						
≤50%	2	1417	2.94 (−1.67; 7.55)	0.212	39.63	0.001
51–80%	11	11180	0.23 (0.01; 0.45)	0.038		
≥ 80%	15	130267	0.06 (0.03; 0.10)	1.2 × 10^-4^		
Adjustment						
Any covariate						
No	13	107239	0.65 (0.16; 1.14)	0.009	5.02	0.090
Yes	15	35625	0.06 (0.02; 0.09)	0.001		
Sex						
No	15	111530	0.50 (0.22; 0.77)	4.3 × 10^-4^	0.00	0.206
Yes	13	31334	0.05 (0.01; 0.09)	0.010		
Age						
No	17	110640	0.48 (0.15; 0.80)	0.004	0.00	0.217
Yes	11	32224	0.06 (0.02; 0.10)	0.002		
Population substructure						
No	19	118203	0.38 (0.17; 0.58)	4.3 × 10^-4^	0.00	0.284
Yes	9	24661	0.04 (0.00; 0.08)	0.066

**Table 6.** Random effects meta-analysis and meta-regression results for the mean difference (β) in BMI comparing LP with non-LP (reference group) individuals, stratifying according to study characteristics, excluding an outlying study

Characteristic	Number of		Meta-analysis		Meta-regression	
	Estimates	Individuals	β (95% CI)	*P*-value	Adjusted-R^2^	*P*-value
Sample size						
64–529	9	3454	0.06 (−0.31; 0.43)	0.754	0.00	0.876
580–2104	9	11788	0.13 (−0.02; 0.28)	0.081		
2126-97811	9	127558	0.08 (0.05; 0.11)	7.1 × 10^-7^		
Age						
9–13	3	1470	0.14 (−0.29; 0.56)	0.529	0.00	0.739
26–55	15	28547	0.06 (0.00; 0.13)	0.038		
56–69	9	112783	0.10 (0.03; 0.18)	0.006		
Ancestry						
European	25	141153	0.08 (0.04; 0.12)	3.7 × 10^-4^	0.00	0.481
Other	2	1647	0.26 (−0.41; 0.93)	0.446		
Continent						
European	23	139392	0.08 (0.03; 0.12)	4.8 × 10^-4^	0.00	0.581
Other	4	3408	0.18 (−0.20; 0.56)	0.345		
LP prevalence						
≤50%	1	1353	0.60 (−0.06; 1.25)	0.074	16.63	0.129
51–80%	11	11180	0.23 (0.01; 0.45)	0.038		
≥ 80%	15	130267	0.06 (0.03; 0.10)	1.2 × 10^-4^		
Adjustment						
Any covariate						
No	12	107175	0.25 (0.11; 0.40)	0.001	64.98	0.011
Yes	15	35625	0.06 (0.02; 0.09)	0.001		
Sex						
No	14	111466	0.16 (0.07; 0.26)	0.001	27.90	0.061
Yes	13	31334	0.05 (0.01; 0.09)	0.010		
Age						
No	16	110576	0.19 (0.05; 0.32)	0.006	15.87	0.104
Yes	11	32224	0.06 (0.02; 0.10)	0.002		
Population substructure						
No	18	118139	0.14 (0.08; 0.21)	2.1 × 10^-5^	59.78	0.017
Yes	9	24661	0.04 (0.00; 0.08)	0.067		

### Power analysis

Supplementary Tables 9 and 10 (available as Supplementary data at *IJE* online) display the expected results of the LP-blood pressure associations assuming that they are entirely mediated by BMI. Even in a sample size of 300 000 individuals, statistical power was low, ranging from 5% to 49% for systolic and 5% to 40% for diastolic blood pressure, depending on LP prevalence and strength of LP-BMI associations. In this sample size, the point effect estimates ranged from 0.016 to 0.192 and 0.007 to 0.088 mmHg, respectively, comparing LP to non-LP (reference group) individuals.

## Discussion

In this study, the LP SNP rs4988235 was associated with daily milk consumption in a population-based sample in South Brazil. In conventional observational analysis, there was an inverse association of milk intake with obesity and blood pressure. However, Mendelian randomization analyses based on genetically defined LP did not confirm these associations. Moreover, LP was positively associated with BMI and overweight-obesity in our meta-analysis.

The aforementioned meta-analysis^36^ had already detected that LP individuals have higher BMI compared with non-LP subjects. Our systematic literature review identified several additional reports of the LP-BMI association published after this study, as well as earlier reports, thus allowing expansion of this study. Moreover, a recent study on 97 811 Danish individuals failed to detect strong associations of LP with BMI and overweight-obesity.^30^ Our findings confirmed the previously reported positive association of LP with BMI, which was corroborated by an association with overweight-obesity in the same direction. Although the sample size of the Danish study represented 64% and 95% of the pooled sample sizes for BMI and overweight-obesity (respectively), its actual weight in the meta-analysis is lower due to the very low prevalence of lactase non-persistence in this study. Moreover, influence analysis did not indicate that any single study was substantially influencing the pooled estimates and, in the subgroup analyses performed for BMI, all pooled point estimates were directionally consistent. In additional analyses performed using fixed effects, the pooled estimates for BMI and overweight obesity were 0.08 (95% CI: 0.05; 0.11) and 1.09 (1.02; 1.17), respectively, corroborating the findings obtained with random effects. These findings suggest a positive causal effect of milk intake on BMI and overweight-obesity, suggesting that the negative associations observed in conventional observational analysis were due to residual confounding or other limitations of observational studies.

A recent large study in the Danish population (*n* = 98 529) failed to detect an association of LP with blood pressure and hypertension.^37^ These conclusions were not substantially influenced by adding the reports identified in our systematic literature review and the results for the 1982 Pelotas Birth Cohort. Based on our power calculations, sample sizes larger than 300 000 individuals would be required for properly-powered evaluations of the LP-blood pressure association under the assumption that BMI entirely mediates these associations. Although this suggests that our meta-analysis could be underpowered, it also suggests that even moderate blood pressure-lowering effects of milk could overcome any hypertensive effects due to its positive association with BMI and be detectable in our meta-analysis. Although 95% confidence intervals for diastolic and raised blood pressure were wide (due to heterogeneity between estimates), the point estimates were small and inconsistent when comparing systolic and raised blood pressure with diastolic blood pressure. Moreover, all these estimates were based on data for more than 100 000 individuals, and about half of them suggested a negative association whereas the remaining suggested a positive association. Therefore, although our findings are not entirely conclusive regarding whether or not milk intake is causally associated with blood pressure due to potential power issues, they are not supportive of a strong negative effect.

A negative association between milk intake and hypertension has been consistently reported in observational studies, as shown in two meta-analyses of cohort studies: one included five studies, with approximately 45 000 individuals and 15 000 cases of raised blood pressure;^23^ and the other (which observed a dose-response effect) included nine studies, with approximately 57 000 individuals and 15 500 hypertension cases.^24^ Two meta-analyses (including 12 and 14 studies, with 623 and 1306 individuals, respectively^25^^,^^26^) of RCTs suggested that the milk-derived tripeptides isoleucine-proline-proline and valine-proline-proline have blood pressure-lowering effects. Moreover, pooled estimates from 14 trials involving a total of 702 individuals indicated that probiotic fermented milk has effects on blood pressure in the same direction^56^ when compared with placebo.

In spite of the aforementioned studies, the strength of the evidence supporting a beneficial effect of milk intake on blood pressure is considered moderate.^57^ Findings from RCTs of specific milk compounds (e.g. tripeptides) may not be directly transposable into a feasible public health intervention such as stimulating milk consumption, for several reasons. For example, intake of milk of higher fat content may predominate over lower fat milk in a given population, therefore contributing to greater fat intake. Moreover, RCTs assessing dietary habits are often limited to short time periods and to specific nutritional compounds that can be delivered via capsules or alternatives that allow double blinding. In contrast, Mendelian randomization (depending on the genetic variant used as proxy) allows the study of lifelong effects of a given dietary item as a whole on the outcome: in the case of rs4988235, it is known that lactase levels decrease markedly after weaning in CC individuals.^1^ Furthermore, this design reveals the effects of the exposure to be targeted by public health interventions in real populations, rather than specific compounds in selected sub-populations who are willing to participate in a RCT.

One of the main potential limitations of our study (which is inherent in the Mendelian randomization design) is the assumption of no effects of the genetic variant on the outcome other than through the exposure of interest (i.e. no horizontal pleiotropy). In this regard, we used a genetic variant with strong functional evidence supporting that its phenotypic implications are mediated by milk consumption due to LP status. We tested the association of rs4988235 with intake of different dairy products and observed reliable associations only with milk. Moreover, a recent study on almost 100 000 Danish individuals failed to detect associations with intake of fruit, vegetables, fish, fast food and soda drinks after correcting for multiple testing.^37^ These results suggest that the effect of this SNP on milk consumption is disentangled from other dietary habits. This is an important consideration, given that milk intake is often part of wider dietary patterns,^58–60^ which can distort observational associations. In our study, reliable associations were also not detected with sociodemographic, perinatal, lifestyle or biological measures, except for smoking status. This latter association was not detected in one of the aforementioned large Danish studies,^37^ raising the possibility that the finding in our study was due to multiple testing.

For some exposures such as alcohol intake, it is possible to perform Mendelian randomization stratifying by never vs ever experimenters to gain insights into potential horizontal pleiotropic effects. This is based on the notion that the effect of genetic variants would be on continuation/addition rather than initiation, so one would expect to see associations only among ever experimenters unless the genetic instruments are associated with the outcome through pathways not mediated by alcohol intake.^61^ In the 1982 Pelotas Birth cohort, stratifying by milk intake yielded larger effect magnitudes among non-milk drinkers (although all interaction test *P*-values were > 0.20). Although this is in accordance with rs4988235 having pleiotropic effects, it is important to note that it was not possible to stratify in never vs ever milk drinkers since this information was not available and, even if it were, the prevalence of never milk drinkers after weaning would most likely be very low. We stratified based on current milk intake status, so it is possible that some individuals stopped drinking milk due to LP-associated symptoms. By conditioning on a potential mediator, we might not only bias downwards the effect estimates associated with LP status, but also introduce collider bias.^62^ Indeed, such adjustment may have created a statistical association between LP and alcohol intake among non-milk drinkers, although there was no strong statistical evidence supporting different associations with LP between milk intake strata for the remaining covariates. Moreover, the aforementioned Danish studies did not detect reliable LP-milk intake interactions regarding overweight-obesity^30^ or blood pressure. However, such interactions were detected regarding sex (likely explained by collider bias since milk intake is associated with sex and, conceptually, neither genetically defined LP nor milk intake can influence sex determination) and other covariates.^37^

Another potential source of confounding in Mendelian randomization studies is population substructure, which was reported to interfere with associations of rs4988235 with height in European American populations^63^ and with several outcomes in Britain.^19^ In the 1982 Pelotas Birth Cohort, such confounding could be expected to be even more pronounced due to multi-ethnicity and ethnic-related socioeconomic inequities that exist in this population.^64^ To control for this effect, we explored genome-wide genotyping data to calculate genomic ancestry to be used as covariates. This adjustment eliminated the strong statistical association between genetically defined LP and socioeconomic variables observed in unadjusted analyses, which would be expected to occur due to population stratification. Regarding the meta-analysis, adjusting for population substructure accounted for 60% of the heterogeneity of LP-BMI estimates after excluding a single outlying study (but not when this study–which fulfilled pre-defined inclusion criteria–was included), with adjusted estimates being lower than unadjusted ones. However, the LP-BMI association in the 1982 Pelotas Birth Cohort was strengthened, rather than weakened, once population stratification was adjusted for. Moreover, most of the studies were performed in ethnically homogeneous populations, and the estimates adjusted for other covariates (i.e. sex and age) were similarly larger than their unadjusted counterparts. This suggests that the difference in the magnitudes of estimates adjusted and unadjusted for population stratification might be related to contextual factors (e.g. characteristics of studies that performed multivariable analyses) rather than residual confounding in unadjusted estimates.

It has been suggested that cultural factors associated with acceptance and generality of milk intake influence the association between the latter and genetically defined LP.^19^^,^^20^ Bergholdt and colleagues,^37^ based on data from six European countries, observed that in general the difference in milk intake comparing LP vs non-LP individuals was larger in countries with higher mean levels of milk intake. This is a potential explanation for the stronger association between LP and milk intake in individuals with at least 85% of European genomic ancestry compared with others in the 1982 Pelotas Birth Cohort, because the latter are, on average, poorer, and socioeconomic position was positively associated with dairy intake in this study. Although this complicates interpreting our meta-analytical findings in units of milk intake, this provides an opportunity to explore potential horizontal pleiotropic effects of the rs4988235 variant. This is because the association of LP with health outcomes–comparing populations where the LP-milk intake association is stronger with populations where this association is weaker–would be expected (in case of no horizontal pleiotropy) to be either stronger in the first (if milk intake has causal effects on the studied outcome) or similar and weak in both (in the case of no causal effects). It was not possible to perform such analysis in the present study due to unavailability of the LP-milk intake association in some studies, as well as to heterogeneity in milk intake measurement.

Another potential limitation is the use of a single genetic predictor of LP instead of including other variants at the *MCM6* region. This is of special importance given that 26 haplotypes were derived from 10 LP-related SNPs in Southern (European ancestry: *n* = 321; African ancestry: *n* = 182), North Eastern (*n* = 262) and Northern (*n* = 200) Brazilian individuals,^10^ corroborating the notion that genetic variants other than rs4988235 should be considered when studying genetically defined LP in non-European populations.^65^ However, the most polymorphic variants (rs4988235 and rs182549) were in high linkage disequilibrium–similarly to the present study (data not shown). Moreover, only nine individuals were heterozygous for the remaining variants (rs4988234, rs145946881, rs4988233 and -13779 G > C–not included in dbSNP) in the *MCM6* region. Northern individuals presented the highest variability, whereas Southern individuals (the most similar to the 1982 Pelotas Birth Cohort) presented one case of heterozygozity for each of the rs145946881 and -13779 G > C variants. Importantly, *in vitro* effects on gene expression are available for the former only.^66^ Therefore, although genotyping variants other than rs4988235 may be important for diagnostic purposes, it is unlikely to have substantial impacts on epidemiological investigations, especially in samples of high European ancestry (such as in South Brazil^67^) and when estimating the prevalence of LP is not the primary goal. Although data for non-Europeans were included in the meta-analysis, most of the estimates were generated in individuals of European ancestry. Moreover, it was necessary to focus on a single (rather than several) genetic variant to improve between-study comparability, given the unavailability of LP-milk intake associations in comparable milk intake scales (e.g. mean difference in ml/day comparing LP and non-LP individuals) across studies (as mentioned above).

Considering that obesity and elevated blood pressure are important risk factors for cardiovascular diseases, identifying their determinants is critical for efficient public health interventions aimed at reducing the burden of disease at the population level. In our study, analyses based on genetically defined LP suggested that simply increasing milk intake in the target population would likely increase its BMI and prevalence of overweight-obesity and, at best, not influence blood pressure. Our findings are in accordance with the notion that the results from RCTs evaluating pressure-lowering effects of milk compounds do not imply that simply increasing milk intake in the general population would promote the expected health benefits.

## Funding

This work was supported by different agencies. From 2004 to 2013, the Wellcome Trust supported the 1982 Pelotas Birth Cohort Study. Additional funding was granted from the Brazilian National Research Council (CNPq) and Rio Grande do Sul State Research Support Foundation (FAPERGS). Previous phases of the study were supported by the International Development Research Center, World Health Organization, Overseas Development Administration, European Union, National Support Program for Centers of Excellence (PRONEX) and the Brazilian Ministry of Health.

## Supplementary Material

Supplementary Data
