# Supplementary material for: Association of lactase persistence genotype with milk consumption, obesity and blood pressure: a Mendelian randomization study in the 1982 Pelotas (Brazil) Birth Cohort, with a systematic review and meta-analysis
Source: Int J Epidemiol. 2016 May 11;45(5):1573–87. doi: 10.1093/ije/dyw074 (PMC5100608; doi:10.1093/ije/dyw074)
Supplement: Supplementary Data [file dyw074_supplementary_data.zip › ije-2015-06-0770-File013.docx]

**Supplementary Table 5.** Associations between household asset index quintiles, schooling and daily consumption of dairy products. Values are number of subjects (N), means (95% confidence intervals) and P-values.

| **Socioeconomic** | **Milk (ml)** | **Yogurt (ml)** | **Cheese (g)** | **Cottage cheese (g)** |
| --- | --- | --- | --- | --- |
| **Variable** |  |  |  |  |
| **Household asset index** | P=0.013 | P=0.048 | P=2.7×10^-16^ | P=2.9×10^-7^ |
| 1^st^ (Poorest) (N=620) | 184 (159; 208) | 60 (48; 73) | 7.4 (6.2; 8.6) | 1.5 (1.1; 1.9) |
| 2^nd^ (N=559) | 179 (155; 202) | 70 (56; 83) | 10.2 (8.7; 11.6) | 1.9 (1.4; 2.4) |
| 3^rd^ (N=698) | 210 (187; 232) | 81 (68; 94) | 11.6 (10.4; 12.8) | 2.5 (2.0; 3.0) |
| 4^th^ (N=271) | 225 (186; 265) | 75 (61; 90) | 13.3 (11.0; 15.5) | 2.3 (1.8; 2.9) |
| 5^th^ (Richest) (N=510) | 234 (205; 263) | 87 (74; 100) | 16.2 (14.5; 17.8) | 3.8 (3.0; 4.6) |
| **Achieved schooling** | P=1.3×10^-4^ | P=0.102 | P=2.0×10^-13^ | P=7.9×10^-7^ |
| 0-4 years (N=171) | 193 (142; 245) | 63 (32; 94) | 8.5 (5.8; 11.2) | 1.7 (0.9; 2.5) |
| 5-8 years (N=539) | 164 (140; 188) | 63 (50; 76) | 7.4 (6.2; 8.6) | 1.8 (1.2; 2.3) |
| 9-11 years (N=859) | 192 (172; 212) | 74 (61; 87) | 10.7 (9.4; 11.9) | 1.7 (1.4; 2.0) |
| ≥12 years (N=1228) | 233 (215; 251) | 82 (75; 90) | 14.0 (13.0; 15.0) | 3.2 (2.7; 3.7) |

P-values based on analysis of variance with 4 df (asset index) and 3 df (schooling).
