# Supplementary material for: Association of lactase persistence genotype with milk consumption, obesity and blood pressure: a Mendelian randomization study in the 1982 Pelotas (Brazil) Birth Cohort, with a systematic review and meta-analysis
Source: Int J Epidemiol. 2016 May 11;45(5):1573–87. doi: 10.1093/ije/dyw074 (PMC5100608; doi:10.1093/ije/dyw074)
Supplement: Supplementary Data [file dyw074_supplementary_data.zip › ije-2015-06-0770-File014.docx]

**Supplementary Table 6.** rs4988235 frequencies according to self-reported skin colour.

| **Covariates** | **Skin colour** | **Genotypes (%)** | | | **Total (N)** | **P-values** | |
| --- | --- | --- | --- | --- | --- | --- | --- |
|  |  | **C/C** | **C/T** | **T/T** |  | **HWE** | **χ^2^ test** |
| None | White | 37.3 | 47.9 | 14.8 | 2158 | 0.717 | 2.1×10^-36^ |
|  | Brown | 55.3 | 38.5 | 6.2 | 161 | >0.999 |  |
|  | Black | 70.2 | 27.2 | 2.6 | 426 | >0.999 |  |
|  | Other^b^ | 50.0 | 41.8 | 8.2 | 98 | >0.999 |  |
|  | All | 43.7 | 44.1 | 12.3 | 2843 | 0.244 |  |
| African and Native-American genomic ancestry^a^ | White | 44.9 | 46.0 | 9.1 | 2158 | - | 0.983 |
|  | Brown | 43.9 | 45.9 | 10.3 | 161 | - |  |
|  | Black | 42.8 | 44.9 | 12.2 | 426 | - |  |
|  | Other^b^ | 47.2 | 44.9 | 7.9 | 98 | - |  |

N: Number of individuals; HWE: Hardy-Weinberg Equilibrium.

The χ^2^ test is testing the association between self-reported skin colour and rs4988235.

^a^The proportions reported were predicted from a multinomial regression model. The χ^2^ test corresponds to a likelihood ratio test.

^b^Other: Asian (n=53; 54.1%) and Native-American (n=45; 45.9%).
