# Supplementary material for: Association of lactase persistence genotype with milk consumption, obesity and blood pressure: a Mendelian randomization study in the 1982 Pelotas (Brazil) Birth Cohort, with a systematic review and meta-analysis
Source: Int J Epidemiol. 2016 May 11;45(5):1573–87. doi: 10.1093/ije/dyw074 (PMC5100608; doi:10.1093/ije/dyw074)
Supplement: Supplementary Data [file dyw074_supplementary_data.zip › ije-2015-06-0770-File015.docx]

**Supplementary Table 7.** Linear regression coefficients (β) of potential continuous confounders according to rs4988235 genotypes (independent variable; CC=0 and T-allele carriers=1) and odds ratio (OR) of rs4988235 genotypes (dependent variable: CC=0; T-allele carriers=1) according to sociodemographic, perinatal, lifestyle and biological variables.

| **Age at measurement** | **Exposures**^a^ | **Crude** | **Adjusted**^b^ | | |
| --- | --- | --- | --- | --- | --- |
|  |  |  | **Total** | **Non-drinkers** | **Milk drikers** |
| At birth (baseline) | **Sex** |  |  | P_interaction_=0.193 | |
|  |  | P=0.803 | P=0.573 | P=0.183 | P=0.827 |
|  | Males (OR) | 1 (Ref.) | 1 (Ref.) | 1 (Ref.) | 1 (Ref.) |
|  | Females (OR) | 1.0 (0.9; 1.2) | 1.0 (0.9; 1.2) | 1.3 (0.9; 1.8) | 1.0 (0.8; 1.2) |
|  | **Birthweight (g)** |  |  | P_interaction_=0.800 | |
|  |  | P=0.107 | P=0.970 | P=0.727 | P=0.901 |
|  | β (95% CI) | 31 (-7; 69) | -1 (-40; 39) | 17 (-80; 115) | -3 (-46; 40) |
|  | **Gestational age (weeks)** |  |  | P_interaction_=0.189 | |
|  |  | P=0.196 | P=0.572 | P=0.379 | P=0.295 |
|  | β (95% CI) | 0.1 (0.0; 0.2) | 0.0 (-0.1; 0.2) | -0.2 (-0.5; 0.2) | 0.1 (-0.1; 0.3) |
| 22-23 years (2004-2005 follow-up) | **Skin colour** |  |  | P_interaction_=0.452 | |
|  |  | P=2.1×10^-36^ | P=0.935 | P=0.508 | P=0.986 |
|  | White (OR) | 1 (Ref.) | 1 (Ref.) | 1 (Ref.) | 1 (Ref.) |
|  | Brown (OR) | 0.5 (0.3; 0.7) | 1.0 (0.7; 1.5) | 1.1 (0.5; 2.5) | 1.0 (0.6; 1.5) |
|  | Black (OR) | 0.3 (0.2; 0.3) | 1.1 (0.7; 1.6) | 1.2 (0.5; 2.6) | 1.1 (0.7; 1.8) |
|  | Other (OR) | 0.6 (0.4; 0.9) | 0.9 (0.6; 1.4) | 0.6 (0.2; 1.3) | 1.1 (0.6; 1.8) |
|  | **Physical activity (min/week)** |  |  | P_interaction_=0.965 | |
|  |  | P=0.498 | P=0.598 | P=0.929 | P=0.637 |
|  | β (95% CI) | -7 (-26; 12) | -5 (-25; 15) | 2 (-.3; 45) | -5 (-28; 17) |
| 30-31 years (2012-2013 follow-up) | **Achieved schooling (years)** |  |  | P_interaction_=0.300 | |
|  |  | P=0.009 | P=0.346 | P=0.583 | P=0.143 |
|  | β (95% CI) | 0.4 (0.1; 0.7) | -0.1 (-0.5; 0.2) | 0.2 (-0.5; 1.0) | -0.3 (-0.6; 0.1) |
|  | **Household asset index (Z-scores)** |  |  | P_interaction_=0.407 | |
|  |  | P=2.4×10^-4^ | P=0.837 | P=0.467 | P=0.457 |
|  | β (95% CI) | 0.1 (0.1; 0.2) | 0.0 (-0.1; 0.1) | 0.1 (-0.1; 0.2) | 0.0 (-0.1; 0.1) |
|  | **Smoking** |  |  | P_interaction_=0.068 | |
|  |  | P=0.084 | P=0.008 | P=0.483 | P=4.9×10^-4^ |
|  | Never (OR) | 1 (Ref.) | 1 (Ref.) | 1 (Ref.) | 1 (Ref.) |
|  | Ex-smoker (OR) | 1.1 (0.9; 1.3) | 1.1 (0.9; 1.4) | 1.3 (0.8; 2.2) | 1.1 (0.8; 1.3) |
|  | Smoker (OR) | 1.2 (1.0; 1.5) | 1.4 (1.1; 1.6) | 1.0 (0.6; 1.5) | 1.5 (1.2; 1.9) |
|  | **Alcohol intake (g/day)** |  |  | P_interaction_=0.018 | |
|  |  | P=0.386 | P=0.710 | P=0.028 | P=0.432 |
|  | β (95% CI) | -0.4 (-1.5; 0.6) | -0.2 (-1.3; 0.9) | -2.9 (-5.5; -0.3) | 0.5 (-0.7; 1.6) |
|  | **LDL (mg/dl)** |  |  | P_interaction_=0.986 | |
|  |  | P=0.945 | P=0.826 | P=0.975 | P=0.848 |
|  | β (95% CI) | 0.1 (-2.1; 2.3) | -0.3 (-2.6; 2.0) | 0.1 (-5.3; 5.4) | -0.2 (-2.8; 2.3) |
|  | **Height (cm)** |  |  | P_interaction_=0.148 | |
|  |  | P=0.790 | P=0.330 | P=0.071 | P=0.947 |
|  | β (95% CI) | 0.1 (-0.6; 0.8) | -0.4 (-1.1; 0.4) | -1.5 (-3.1; 0.1) | 0.0 (-0.8; 0.8) |

^a^Variables were treated as continuous (dependent variables in linear regression), except sex, skin colour and smoking (independent variables in logistic regression).

^b^Controlled for the African and Native-American genomic ancestry.
