# Supplementary material for: Association of lactase persistence genotype with milk consumption, obesity and blood pressure: a Mendelian randomization study in the 1982 Pelotas (Brazil) Birth Cohort, with a systematic review and meta-analysis
Source: Int J Epidemiol. 2016 May 11;45(5):1573–87. doi: 10.1093/ije/dyw074 (PMC5100608; doi:10.1093/ije/dyw074)
Supplement: Supplementary Data [file dyw074_supplementary_data.zip › ije-2015-06-0770-File016.docx]

**Supplementary Table 8.** Association of genetically-defined LP (reference group: non-LP individuals) with studied outcomes in the 1982 Pelotas (Brazil) Birth Cohort, stratifying by milk intake status (drinkers vs. non-drinkers). Values are number of individuals (N), linear regression coefficients (β) or odds ratio (OR), 95% confidence intervals (95% CI) and P-values (P).

| **Outcome** | **Unadjusted** | **Adjusted for sex and genomic ancestry** | | |
| --- | --- | --- | --- | --- |
|  |  | **All** | **Drinkers** | **Non-drinkers** |
| **BMI (kg/m²)** |  |  | P_interaction_=0.588 | |
| N | 2782 | 2780 | 2226 | 533 |
| P | 0.207 | 0.052 | 0.333 | 0.026 |
| β (95% CI) | 0.27 (-0.15; 0.69) | 0.44 (0.00; 0.88) | 0.24 (-0.24; 0.71) | 1.25 (0.15; 2.35) |
| **Overweight-obesity^a^** |  |  | P_interaction_=0.207 | |
| N | 2782 | 2780 | 2226 | 533 |
| P | 0.565 | 0.294 | 0.850 | 0.039 |
| OR (95% CI) | 1.05 (0.90; 1.22) | 1.09 (0.93; 1.28) | 1.02 (0.85; 1.22) | 1.49 (1.02; 2.19) |
| **Systolic BP (mmHg)** |  |  | P_interaction_=0.790 | |
| N | 2840 | 2838 | 2264 | 540 |
| P | 0.503 | 0.362 | 0.569 | 0.383 |
| β (95% CI) | -0.35 (-1.38; 0.67) | 0.43 (-0.50; 1.37) | 0.30 (-0.74; 1.34) | 0.96 (-1.20; 3.13) |
| **Diastolic BP (mmHg)** |  |  | P_interaction_=0.928 | |
| N | 2840 | 2838 | 2264 | 540 |
| P | 0.773 | 0.467 | 0.665 | 0.511 |
| β (95% CI) | -0.10 (-0.80; 0.60) | 0.27 (-0.45; 0.99) | 0.18 (-0.62; 0.98) | 0.56 (-1.11; 2.23) |
| **Raised BP^b^** |  |  | P_interaction_=0.333 | |
| N | 2840 | 2838 | 2264 | 540 |
| P | 0.496 | 0.684 | 0.986 | 0.252 |
| OR (95% CI) | 0.94 (0.80; 1.11) | 1.04 (0.87; 1.25) | 1.00 (0.81; 1.23) | 1.27 (0.85; 1.90) |

BMI: Body mass index. BP: blood pressure.

^a^BMI≥25 kg/m².

^b^Systolic BP≥130 mmHg or dystolic BP≥85 mmHg.
