# Supplementary material for: Association of lactase persistence genotype with milk consumption, obesity and blood pressure: a Mendelian randomization study in the 1982 Pelotas (Brazil) Birth Cohort, with a systematic review and meta-analysis
Source: Int J Epidemiol. 2016 May 11;45(5):1573–87. doi: 10.1093/ije/dyw074 (PMC5100608; doi:10.1093/ije/dyw074)
Supplement: Supplementary Data [file dyw074_supplementary_data.zip › ije-2015-06-0770-File017.docx]

**Supplementary Table 9.** Linear regression coefficients (β), standard errors (SE) and power of the association of genetically-defined LP (reference group: non-LP individuals) with systolic blood pressure (SBP) – assuming that BMI entirely mediates the association – based on 10,000 simulations per scenario. In all scenarios, standard deviation values of BMI and SBP were 4.4 and 22.8, respectively.

| **N** | **LP prevalence** | **LP-BMI association** | | **BMI-SBP association** | | **LP-SBP association** | | |
| --- | --- | --- | --- | --- | --- | --- | --- | --- |
|  |  | **β** | **SE** | **Β** | **SE** | **Mean β** | **Mean SE** | **Power (%)** |
| 150,000 | 94.06% | 0.079 | 0.015 | 0.70 | 0.235 | 0.018 | 0.249 | 5.1 |
|  | 74.67% | 0.172 | 0.051 |  |  | 0.037 | 0.139 | 6.1 |
|  | 94.06% | 0.079 | 0.015 | 3.85 | 1.007 | 0.086 | 0.249 | 7.3 |
|  | 74.67% | 0.172 | 0.051 |  |  | 0.196 | 0.139 | 31.4 |
| 200,000 | 94.06% | 0.079 | 0.015 | 0.70 | 0.235 | 0.019 | 0.216 | 5.0 |
|  | 74.67% | 0.172 | 0.051 |  |  | 0.035 | 0.120 | 6.9 |
|  | 94.06% | 0.079 | 0.015 | 3.85 | 1.007 | 0.085 | 0.216 | 7.01 |
|  | 74.67% | 0.172 | 0.051 |  |  | 0.193 | 0.120 | 37.6 |
| 250,000 | 94.06% | 0.079 | 0.015 | 0.70 | 0.235 | 0.017 | 0.193 | 5.1 |
|  | 74.67% | 0.172 | 0.051 |  |  | 0.036 | 0.107 | 6.9 |
|  | 94.06% | 0.079 | 0.015 | 3.85 | 1.007 | 0.088 | 0.193 | 7.5 |
|  | 74.67% | 0.172 | 0.051 |  |  | 0.194 | 0.107 | 44.9 |
| 300,000 | 94.06% | 0.079 | 0.015 | 0.70 | 0.235 | 0.016 | 0.176 | 5.0 |
|  | 74.67% | 0.172 | 0.051 |  |  | 0.034 | 0.098 | 6.7 |
|  | 94.06% | 0.079 | 0.015 | 3.85 | 1.007 | 0.087 | 0.176 | 8.5 |
|  | 74.67% | 0.172 | 0.051 |  |  | 0.192 | 0.098 | 49.2 |
