# Supplementary material for: Association of lactase persistence genotype with milk consumption, obesity and blood pressure: a Mendelian randomization study in the 1982 Pelotas (Brazil) Birth Cohort, with a systematic review and meta-analysis
Source: Int J Epidemiol. 2016 May 11;45(5):1573–87. doi: 10.1093/ije/dyw074 (PMC5100608; doi:10.1093/ije/dyw074)
Supplement: Supplementary Data [file dyw074_supplementary_data.zip › ije-2015-06-0770-File018.docx]

**Supplementary Table 10.** Linear regression coefficients (β), standard errors (SE) and power of the association of genetically-defined LP (reference group: non-LP individuals) with diastolic blood pressure (DBP) – assuming that BMI entirely mediates the association – based on 10,000 simulations per scenario. In all scenarios, standard deviation values of BMI and DBP were 4.4 and 12.3, respectively.

| **N** | **LP prevalence** | **LP-BMI association** | | **BMI-DBP association** | | **LP-DBP association** | | |
| --- | --- | --- | --- | --- | --- | --- | --- | --- |
|  |  | **β** | **SE** | **Β** | **SE** | **Mean β** | **Mean SE** | **Power (%)** |
| 150,000 | 94.06% | 0.079 | 0.015 | 0.70 | 0.235 | 0.007 | 0.134 | 5.2 |
|  | 74.67% | 0.172 | 0.051 |  |  | 0.014 | 0.075 | 5.6 |
|  | 94.06% | 0.079 | 0.015 | 3.85 | 1.007 | 0.042 | 0.134 | 5.9 |
|  | 74.67% | 0.172 | 0.051 |  |  | 0.092 | 0.075 | 26.3 |
| 200,000 | 94.06% | 0.079 | 0.015 | 0.70 | 0.235 | 0.004 | 0.116 | 5.0 |
|  | 74.67% | 0.172 | 0.051 |  |  | 0.014 | 0.065 | 5.5 |
|  | 94.06% | 0.079 | 0.015 | 3.85 | 1.007 | 0.038 | 0.116 | 6.5 |
|  | 74.67% | 0.172 | 0.051 |  |  | 0.090 | 0.065 | 31.3 |
| 250,000 | 94.06% | 0.079 | 0.015 | 0.70 | 0.235 | 0.007 | 0.104 | 5.3 |
|  | 74.67% | 0.172 | 0.051 |  |  | 0.014 | 0.058 | 6.1 |
|  | 94.06% | 0.079 | 0.015 | 3.85 | 1.007 | 0.040 | 0.104 | 6.9 |
|  | 74.67% | 0.172 | 0.051 |  |  | 0.089 | 0.058 | 35.6 |
| 300,000 | 94.06% | 0.079 | 0.015 | 0.70 | 0.235 | 0.007 | 0.095 | 5.2 |
|  | 74.67% | 0.172 | 0.051 |  |  | 0.013 | 0.053 | 6.1 |
|  | 94.06% | 0.079 | 0.015 | 3.85 | 1.007 | 0.040 | 0.095 | 7.2 |
|  | 74.67% | 0.172 | 0.051 |  |  | 0.088 | 0.053 | 40.0 |
