# Supplementary material for: Association of lactase persistence genotype with milk consumption, obesity and blood pressure: a Mendelian randomization study in the 1982 Pelotas (Brazil) Birth Cohort, with a systematic review and meta-analysis
Source: Int J Epidemiol. 2016 May 11;45(5):1573–87. doi: 10.1093/ije/dyw074 (PMC5100608; doi:10.1093/ije/dyw074)
Supplement: Supplementary Data [file dyw074_supplementary_data.zip › ije-2015-06-0770-File019.docx]

**Supplementary Figure 1.** Flow diagram of the 1982 Pelotas Birth Cohort (only including the relevant phases for the present study).

**5914 individuals at baseline (1982)**

**3541 individuals with genetic data**

**2843 individuals with genetic and outcome data**

- 53.6% of the cohort at baseline^a^
- 76.8% of the entire 2012-2013 follow-up visit^b^

**2004-2005 follow-up visit**

**22-23 years of age**

- 4297 individuals interviewed
- 282 known deaths
- Follow-up rate: 77.4%^a^

756 without data for the rs4988235 SNP after quality control filters

**2012-2013 follow-up visit**

**30-31 years of age**

- 3701 individuals interviewed
- 325 known deaths
- Follow-up rate: 68.1%^a^

858 without data for the rs4988235 SNP after quality control filters and for any studied outcome (BMI, diastolic or systolic blood pressure)

^a^Individuals known to have died were included both in the numerator and in the denominator.

^b^Individuals known to have died were not considered in this calculation.
