# Supplementary material for: Association of lactase persistence genotype with milk consumption, obesity and blood pressure: a Mendelian randomization study in the 1982 Pelotas (Brazil) Birth Cohort, with a systematic review and meta-analysis
Source: Int J Epidemiol. 2016 May 11;45(5):1573–87. doi: 10.1093/ije/dyw074 (PMC5100608; doi:10.1093/ije/dyw074)
Supplement: Supplementary Data [file dyw074_supplementary_data.zip › ije-2015-06-0770-File020.docx]

**Supplementary Figure 2.** Flow diagram of study selection.

**611 records identified**

**452 records screened**

150 duplicates removed (128 by OVID and 31 manually identified)

**30 full-texts assessed for eligibility**

- 23 journal articles
- 6 conference abstracts
- 1 collection of conference abstracts

422 titles & abstracts removed

14 removed

- 6 conference abstracts also published as selected journal articles
- 5 journal articles and 1 collection of conference abstracts did not meet inclusion criteria

**16 eligible records**

1 additional eligible journal article found in reference lists

**17 records included**
