# Supplementary material for: Association of lactase persistence genotype with milk consumption, obesity and blood pressure: a Mendelian randomization study in the 1982 Pelotas (Brazil) Birth Cohort, with a systematic review and meta-analysis
Source: Int J Epidemiol. 2016 May 11;45(5):1573–87. doi: 10.1093/ije/dyw074 (PMC5100608; doi:10.1093/ije/dyw074)

**Supplementary Figure 5.** Forest and funnel plots of odds of overweight-obesity comparing LP with non-LP individuals (reference group) based on random effects meta-analysis.


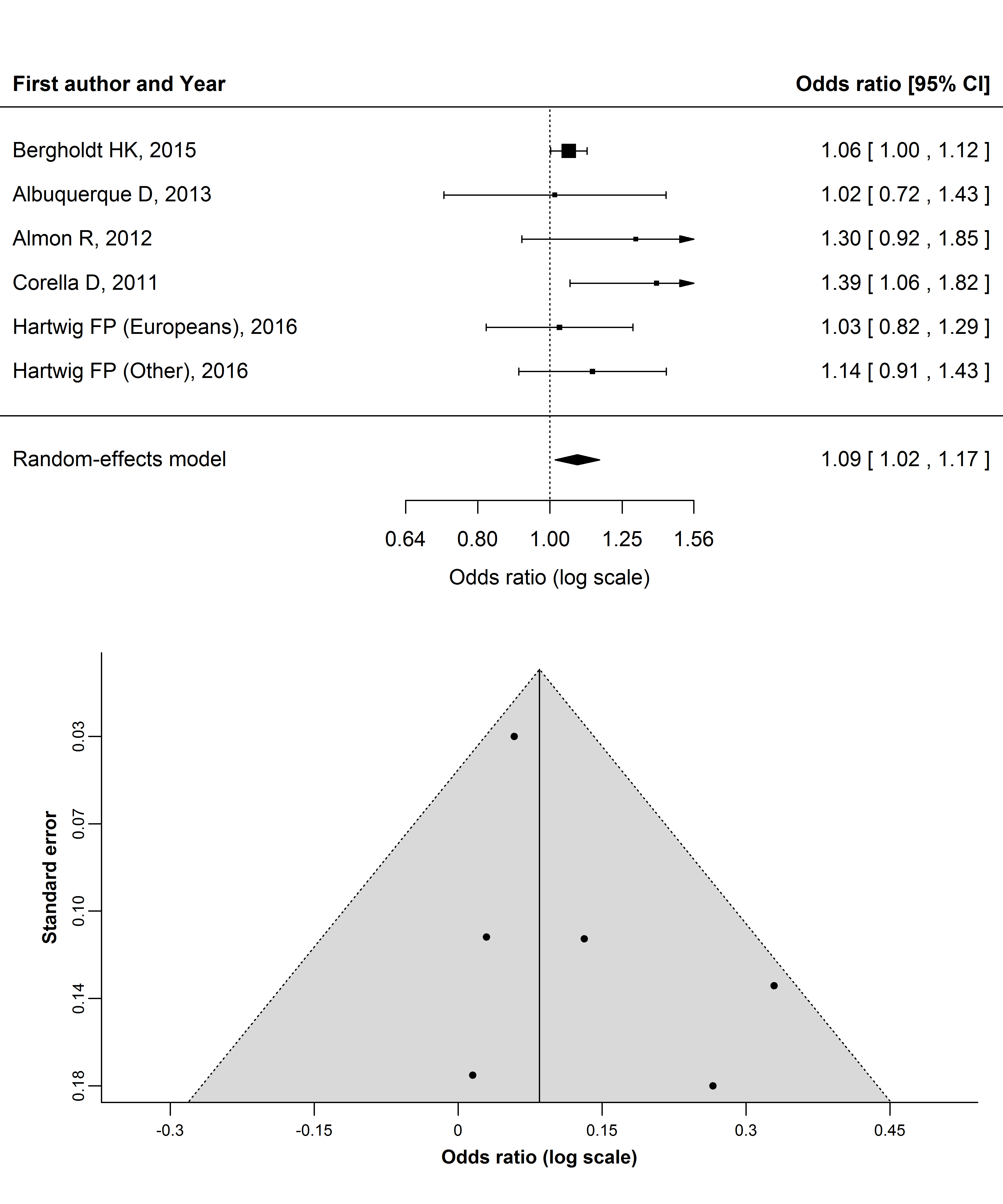

Supplement: Supplementary Data [file dyw074_supplementary_data.zip › ije-2015-06-0770-File023.docx]
