# Supplementary material for: Association of lactase persistence genotype with milk consumption, obesity and blood pressure: a Mendelian randomization study in the 1982 Pelotas (Brazil) Birth Cohort, with a systematic review and meta-analysis
Source: Int J Epidemiol. 2016 May 11;45(5):1573–87. doi: 10.1093/ije/dyw074 (PMC5100608; doi:10.1093/ije/dyw074)
Supplement: Supplementary Data [file dyw074_supplementary_data.zip › ije-2015-06-0770-File008.docx]

**Association of lactase persistence genotype with milk consumption, obesity and blood pressure: a Mendelian randomization study in the 1982 Pelotas (Brazil) Birth Cohort with a systematic review and meta-analysis**

**SUPPLEMENTARY METHODS**

**Power calculations**

Post hoc power calculations through simulations were performed to evaluate our meta-analysis power to detect associations of LP with systolic and diastolic blood pressure assuming that they are entirely mediated by BMI. Standard deviations of BMI, systolic and diastolic blood pressure were obtained from the aforementioned large Danish study (1) – the largest study included in our meta-analysis – based on the interquartile range using the normal approximation approach described above. The values were 4.4, 22.8 and 12.3, respectively. These were similar compared to one of the studies used to obtain BMI-blood pressure linear regression coefficients and standard errors (described below), which was performed in a sub-sample of the Danish study (2). Sample sizes were 150,000, 200,000, 250,000 and 300000, and BMI was forced to have a minimum value of 15 to avoid negative numbers because, in some simulations, BMI was log-transformed (see below). To account for the uncertainty in the LP-BMI and BMI-blood pressure associations, the actual values used in each simulation were sampled from a normal distribution with mean equal to the point effect estimate and standard deviation equal to the standard error of the point effect estimate.

Two sets of values for LP prevalence, LP-BMI linear regression coefficients (in kg/m², comparing LP with non-LP [reference group] individuals) and standard errors were used: i) 94.06%, 0.079 and 0.015, respectively; ii) 76.47%, 0.172 and 0.051, respectively. Sets i) and ii) corresponds to our fixed and random effects meta-analytical findings, respectively (even though our main analyses used random effects only, we included simulations using estimates based on both models to increase the number of simulated scenarios). LP prevalence corresponds to a weighted estimate based on the weights used in each meta-analysis model (in order to use the same weights used to obtain the pooled effect estimates) for systolic blood pressure.

BMI-blood pressure associations were obtained from two Mendelian randomization studies. Two sets of values for BMI-systolic and BMI-diastolic blood pressure linear regression coefficients (standard errors) were used: i) 0.70 (0.235) and 0.28 (0.125), respectively; ii) 3.85 (1.007) and 1.79 (0.566), respectively. Set i) corresponds to linear regression coefficients and standard errors of blood pressure (mmHg) per 1 kg/m² increase in BMI based on six population-based European cohorts (n=30,136). Estimates were obtained using two-stage least-squares regression, having an allelic score based on 14 BMI-associated SNPs as the instrumental variable (3). Set ii) corresponds to linear regression coefficients and standard errors of blood pressure (mmHg) per 10% increase in BMI from an analysis in 36,851 participants in the Copenhagen General Population Study. Estimates were obtained using the generalized method of moments with robust standard errors, using both rs9939609 and rs17782313 (in *FTO* and *MC4R* genes, respectively) as instruments (2). Therefore, for simulations using set ii), BMI was transformed as follows: $\left( \text{log}_{\text{10}} \text{(}\text{BMI}\text{)} \right)\text{×23.03}$.

Combining the two sets of values for LP prevalence and LP-BMI associations and the two sets of values for BMI-blood pressure associations yielded four distinct scenarios for each sample size – totalizing 16 scenarios. For each scenario, mean LP-blood pressure linear regression coefficients and standard errors, as well as power to detect these associations, based on 10,000 simulations were reported. Power was defined as the proportion of simulations that presented a two-sided T-test P-value <0.05.

**REFERENCES**

1. Bergholdt HK, Nordestgaard BG, Ellervik C. Milk intake is not associated with low risk of diabetes or overweight-obesity: a Mendelian randomization study in 97,811 Danish individuals. Am J Clin Nutr 2015; 102(2):487-496.

2. Timpson NJ, Harbord R, Davey Smith G, Zacho J, Tybjaerg-Hansen A, Nordestgaard BG. Does greater adiposity increase blood pressure and hypertension risk?: Mendelian randomization using the FTO/MC4R genotype. Hypertension 2009; 54(1):84-90.

3. Holmes MV, Lange LA, Palmer T, Lanktree MB, North KE, Almoguera B, et al. Causal effects of body mass index on cardiometabolic traits and events: a Mendelian randomization analysis. Am J Hum Genet 2014; 94(2):198-208.
