# Supplementary material for: Association of lactase persistence genotype with milk consumption, obesity and blood pressure: a Mendelian randomization study in the 1982 Pelotas (Brazil) Birth Cohort, with a systematic review and meta-analysis
Source: Int J Epidemiol. 2016 May 11;45(5):1573–87. doi: 10.1093/ije/dyw074 (PMC5100608; doi:10.1093/ije/dyw074)
Supplement: Supplementary Data [file dyw074_supplementary_data.zip › ije-2015-06-0770-File009.docx]

**Supplementary Table 1.** Search terms used for retrieving studies pertaining to each topic.

| **Topic** | **Search terms** |
| --- | --- |
| LP | ("rs4988235$" OR "13910$" OR ("lactase persistence" AND ("genotype?" OR "genetic" OR "allele?"))) |
| BMI | ("body mass index" OR "BMI" OR "obes$").af. |
| Blood pressure | ("systolic" OR "diastolic" OR "blood pressure" OR "arterial pressure" OR "hypertens$").af. |
| Other | ("fat$" OR "adipos$" OR "anthropometr$" OR "cardiovascular" OR "vascular" OR "metabolic" OR "health") |

Using “$” retrieves any number (including zero) of characters after the stem word (eg, obes$ retrieves “obesity”, “obese”, etc).

Using “.af.” allows simultaneously searching all searchable fields in the database. By default, Ovid searches the following fields (some of which are database-specific) when all of its databases are searched: Title, Original Title, Title Comment, Abstract, Subject Heading Word, MeSH Subject Headings, Keyword Heading, Keyword Heading Word, Key Concepts, Full Text, Cited Reference Author Word and others.
