# Supplementary material for: Association of lactase persistence genotype with milk consumption, obesity and blood pressure: a Mendelian randomization study in the 1982 Pelotas (Brazil) Birth Cohort, with a systematic review and meta-analysis
Source: Int J Epidemiol. 2016 May 11;45(5):1573–87. doi: 10.1093/ije/dyw074 (PMC5100608; doi:10.1093/ije/dyw074)
Supplement: Supplementary Data [file dyw074_supplementary_data.zip › ije-2015-06-0770-File010.docx]

**Supplementary Table 2.** Number of results retrieved (December 9, 2015).

| **Search strategy** | **Number of records** |
| --- | --- |
| LP | 2355 |
| BMI | 1726234 |
| Blood pressure | 3059254 |
| Other | 15169435 |
| LP AND (BMI OR Blood pressure OR Other) | 483 |
