# Supplementary material for: Association of lactase persistence genotype with milk consumption, obesity and blood pressure: a Mendelian randomization study in the 1982 Pelotas (Brazil) Birth Cohort, with a systematic review and meta-analysis
Source: Int J Epidemiol. 2016 May 11;45(5):1573–87. doi: 10.1093/ije/dyw074 (PMC5100608; doi:10.1093/ije/dyw074)
Supplement: Supplementary Data [file dyw074_supplementary_data.zip › ije-2015-06-0770-File011.docx]

**Supplementary Table 3.** Characteristics of the studied individuals (overall and stratified by milk intake status). Values are percentages for categorical variables, and mean and standard deviation for continuous variables.

| **Age at measurement** | **Variables** | **Total** | **Milk intake status** | | **P-value^a^** |
| --- | --- | --- | --- | --- | --- |
|  |  |  | **Non-drinkers** | **Drinkers** |  |
| At birth (baseline) | **Sex** |  |  |  |  |
|  | Males | 48.1 | 42.2 | 49.3 | 0.004 |
|  | Females | 51.9 | 57.8 | 50.7 |  |
|  | **Maternal schooling at birth (years)^b^** |  |  |  |  |
|  | 0-4 | 32.4 | 38.7 | 30.8 | 4.7×10^-4^ |
|  | 5-8 | 43.7 | 43.1 | 43.8 |  |
|  | 9-11 | 11.1 | 8.3 | 11.7 |  |
|  | ≥12 | 12.8 | 9.9 | 13.7 |  |
|  | **Family income at birth (minimum wages)^b^** |  |  |  |  |
|  | ≤1.0 | 19.4 | 24.3 | 18.1 | 0.004 |
|  | 1.1-3.0 | 50.4 | 50.9 | 50.4 |  |
|  | 3.1-6.0 | 19.7 | 17.0 | 20.3 |  |
|  | 6.1-10 | 5.7 | 4.1 | 6.0 |  |
|  | >10 | 4.8 | 3.7 | 5.2 |  |
|  | **Birthweight (g)** |  |  |  |  |
|  | Mean (SD) | 3233 (511) | 3187 (555) | 3245 (498) | 0.025 |
|  | **Gestational age (weeks)**^b^ |  |  |  |  |
|  | Mean (SD) | 39.4 (1.8) | 39.2 (1.9) | 39.4 (1.7) | 0.030 |
| 22-23 years (2004-2005 follow-up) | **Self-reported skin colour** |  |  |  |  |
|  | White | 75.9 | 68.0 | 77.7 | 4.4×10^-5^ |
|  | Brown | 5.7 | 7.4 | 5.3 |  |
|  | Black | 15.0 | 19.6 | 13.8 |  |
|  | Other^c^ | 3.4 | 5.0 | 3.2 |  |
|  | **European genomic ancestry (%)**^b^ |  |  |  |  |
|  | Mean (SD) | 78.1 (19.5) | 74.5 (21.2) | 78.9 (18.9) | 8.6×10^-6^ |
|  | **African genomic ancestry (%)**^b^ |  |  |  |  |
|  | Mean (SD) | 14.7 (18.3) | 17.7 (20.1) | 14 (17.8) | 8.4×10^-5^ |
|  | **Native-American genomic ancestry (%)**^b^ |  |  |  |  |
|  | Mean (SD) | 7.2 (4.6) | 7.8 (5.2) | 7.1 (4.5) | 0.003 |
|  | **Leisure-time physical activity** |  |  |  |  |
|  | 0 min/week | 46.9 | 50.2 | 46.3 | 0.070 |
|  | 1-149 min/week | 23.3 | 24.3 | 23.1 |  |
|  | ≥150 min/week | 29.8 | 25.5 | 30.6 |  |
| 30-31 years (2012-2013 follow-up) | **Achieved schooling (complete years)**^b^ |  |  |  |  |
|  | 1-4 | 6.1 | 8.7 | 5.5 | 1.1×10^-4^ |
|  | 5-8 | 19.3 | 24.1 | 18.1 |  |
|  | 9-11 | 30.7 | 28.6 | 31.2 |  |
|  | ≥12 | 43.9 | 38.6 | 45.2 |  |
|  | **Household asset index (quintiles)**^b^ |  |  |  |  |
|  | Poorest | 23.4 | 28.5 | 22.1 | 0.030 |
|  | 2^nd^ | 21.0 | 20.9 | 21.1 |  |
|  | 3^rd^ | 26.3 | 23.8 | 26.8 |  |
|  | 4^th^ | 10.2 | 10.0 | 10.2 |  |
|  | Richest | 19.1 | 16.8 | 19.8 |  |
|  | **Smoking^b^** |  |  |  |  |
|  | Never | 58.5 | 58.0 | 58.8 | 0.177 |
|  | Ex-smoker | 17.7 | 15.7 | 18.2 |  |
|  | Smoker | 23.8 | 26.3 | 23.0 |  |
|  | **Alcohol intake (g/day)^b^** |  |  |  |  |
|  | 0 | 37.8 | 38 | 37.8 | 0.461 |
|  | 0.1-9.9 | 49.9 | 48.9 | 50.2 |  |
|  | 10-19.9 | 8.6 | 8.3 | 8.6 |  |
|  | ≥20 | 3.7 | 4.8 | 3.4 |  |
|  | **LDL (mg/dl)^b^** |  |  |  |  |
|  | Mean (SD) | 109.6 (29.3) | 109.1 (30) | 109.8 (29.2) | 0.631 |
|  | **Body mass index (kg/m²)**^b^ |  |  |  |  |
|  | Mean (SD) | 27.0 (5.6) | 28.1 (6.3) | 26.7 (5.4) | 1.7×10^-6^ |
|  | **Height (cm)**^b^ |  |  |  |  |
|  | Mean (SD) | 168 (9) | 166 (9) | 168 (9) | 4.9×10^-5^ |
|  | **Systolic pressure (mmHg)**^b^ |  |  |  |  |
|  | Mean (SD) | 121.2 (13.8) | 121.8 (14) | 121 (13.7) | 0.237 |
|  | **Diastolic pressure (mmHg)**^b^ |  |  |  |  |
|  | Mean (SD) | 75.6 (9.4) | 76.4 (9.7) | 75.3 (9.3) | 0.022 |
|  | **Milk intake (ml/day)**^b^ |  |  |  |  |
|  | Mean (SD) | 204 (312) | 0 (0) | 253 (329) | 9.2×10^-231^ |
|  | **Yogurt intake (ml/day)**^b^ |  |  |  |  |
|  | Mean (SD) | 75 (164) | 46 (123) | 82 (171) | 2.3×10^-8^ |
|  | **Cheese intake (g/day)**^b^ |  |  |  |  |
|  | Mean (SD) | 11.4 (17.5) | 11.2 (21.4) | 11.4 (16.5) | 0.860 |
|  | **Cottage cheese intake (g/day)**^b^ |  |  |  |  |
|  | Mean (SD) | 2.4 (6.9) | 1.6 (4.6) | 2.6 (7.3) | 7.4×10^-5^ |
|  | **Number of individuals** | 2843 | 540^d^ | 2268^d^ |  |

Only individuals with data for rs4988235 and at least one studied outcome (BMI, systolic or diastolic blood pressure) were included in the present study.

SD: Standard deviation. IQR: Interquartile range.

^a^P-values based on χ^2^ test (for categorical variables), or T-test (for continuous variables).

^b^Variables with missing observations. The largest numbers were 557 (19.6%), 177 (6.2%), 70 (2.5%), 61 (2.1%) and 38 (1.3%), observed in gestational age, household asset index, LDL, body mass index and achieved schooling, respectively.

^c^Other: Asian (n=53; 54.1%) and Native-American (n=45; 45.9%).

^d^These numbers add to 2808 due to 35 individuals with missing information for milk intake.
