# Supplementary material for: Association of lactase persistence genotype with milk consumption, obesity and blood pressure: a Mendelian randomization study in the 1982 Pelotas (Brazil) Birth Cohort, with a systematic review and meta-analysis
Source: Int J Epidemiol. 2016 May 11;45(5):1573–87. doi: 10.1093/ije/dyw074 (PMC5100608; doi:10.1093/ije/dyw074)
Supplement: Supplementary Data [file dyw074_supplementary_data.zip › ije-2015-06-0770-File012.docx]

**Supplementary Table 4.** Comparison of the whole cohort at baseline and of the entire 2012-2013 years of age follow-up visit with those included in the present study. Values are number of subjects and percentages.

| **Comparison group** | **Variable** | **% studied** | **P-value** |
| --- | --- | --- | --- |
| Baseline^a^ | **Sex** |  |  |
|  | Males | 51.4 | 0.001 |
|  | Females | 55.8 |  |
|  | **Maternal schooling at birth (years)** |  |  |
|  | 0-4 | 54.2 | 1.8×10^-6^ |
|  | 5-8 | 56.1 |  |
|  | 9-11 | 52.4 |  |
|  | ≥12 | 45.4 |  |
|  | **Family income at birth (minimum wages)** |  |  |
|  | ≤1.0 | 51.9 | 5.4×10^-7^ |
|  | 1.1-3.0 | 56.3 |  |
|  | 3.1-6.0 | 54.2 |  |
|  | 6.1-10 | 45.0 |  |
|  | >10 | 43.6 |  |
|  | **Birthweight (g)** |  |  |
|  | ≥2500 | 53.4 | 0.389 |
|  | <2500 | 55.4 |  |
|  | **Overall** | 53.6 |  |
| 2012-2013 follow-up^b^ | **Achieved schooling (complete years)** |  |  |
|  | 1-4 | 76.7 | 0.219 |
|  | 5-8 | 74.7 |  |
|  | 9-11 | 78.9 |  |
|  | ≥12 | 77.1 |  |
|  | **Household asset index (quintiles)** |  |  |
|  | 1^st^ quintile (poorest) | 77.2 | 0.062 |
|  | 2^nd^ quintile | 77.1 |  |
|  | 3^rd^ quintile | 78.3 |  |
|  | 4^th^ quintile | 82.6 |  |
|  | 5^th^ quintile (wealthiest) | 74.5 |  |
|  | **BMI (kg/m²) – 30 years** |  |  |
|  | <25 | 77.3 | 0.018 |
|  | 25-29.9 | 77.2 |  |
|  | ≥30 | 81.9 |  |
|  | **Overall** | 76.8 |  |

BMI: body mass index.

Only individuals with data for rs4988235 and at least one studied outcome (BMI, systolic or diastolic blood pressure) were included in the present study.

^a^Individuals known to have died were included both in the numerator and in the denominator.

^b^Individuals known to have died were not considered in this calculation.
